# Family Caregiver Assessment in Primary Care: A Nationwide Survey

**DOI:** 10.1093/geroni/igaa057.239

**Published:** 2020-12-16

**Authors:** Catherine Riffin, Jennifer Wolff, Julianna Maisano, Sylvia Lee, Karl Pillemer

**Affiliations:** 1 Weill Cornell Medical College, London, England, United Kingdom; 2 Johns Hopkins Bloomberg School of Public Health, Baltimore, Maryland, United States; 3 Weill Cornell Medicine, New York City, New York, United States; 4 Weill Cornell Medicine, New York, New York, United States; 5 Cornell University, Ithaca, New York, United States

## Abstract

Family caregivers play an important role in the healthcare of older adults, but their circumstances, needs, and risks are often unknown to medical professionals. Standardizing how caregivers’ needs are assessed in healthcare delivery can help clinicians design care plans that take caregivers’ capabilities into account and provide targeted recommendations for caregiver support. Despite the potential of caregiver assessment, little is known about its use in primary care practice. The present study surveyed a national random sample of 1,000 U.S. primary care clinicians (physicians, nurses, social workers) to characterize current practices, barriers, and facilitators of caregiver assessment. A total of 231 completed responses were received. A minority of respondents (11%) reported that their practice or clinic had a standardized procedure for caregiver assessment; one in ten (10%) reported that they had personally conducted a caregiver assessment using a standardized instrument in the past year. The most common barriers to caregiver assessment were lack of time (65%), inability to have private discussions with caregivers (36%), lack of access to referral options (30%), inadequate reimbursement (30%), and reluctance of caregivers to discuss their needs (30%). The most frequently endorsed facilitators to aid future implementation included better availability of referral options (77%), easier referral mechanisms (67%), co-location of mental health specialists, care managers, or social workers (65%), and training in how to address caregiver issues (61%). Findings are discussed within the context of emerging healthcare policies and practice initiatives designed to promote caregiver assessment in health care settings.

